# Age-associated changes in basal NF-κB function in human CD4^+^ T lymphocytes via dysregulation of PI3 kinase

**DOI:** 10.18632/aging.100705

**Published:** 2014-12-06

**Authors:** Arsun Bektas, Yongqing Zhang, Elin Lehmann, William H. Wood, Kevin G. Becker, Karen Madara, Luigi Ferrucci, Ranjan Sen

**Affiliations:** ^1^ Translational Gerontology Branch, National Institute on Aging, Baltimore, MD 21224, USA; ^2^ Laboratory of Molecular Biology and Immunology, National Institute on Aging, Baltimore, MD 21224, USA; ^3^ Laboratory of Genetics, National Institute on Aging, Baltimore, MD 21224, USA; ^4^ Clinical Research Branch, National Institute on Aging, Baltimore, MD 21224, USA

**Keywords:** CD4+ T cells, NF-κB, PI3K, human aging, gene expression

## Abstract

Immune impairment and high circulating level of pro-inflammatory cytokines are landmarks of human aging. However, the molecular basis of immune dys-regulation and the source of inflammatory markers remain unclear. Here we demonstrate that in the absence of overt cell stimulation gene expression mediated by the transcription factor NF-κB is higher in purified and rested human CD4^+^ T lymphocytes from older compared to younger individuals. This increase of NF-κB-associated transcription includes transcripts for pro-inflammatory cytokines such as IL-1 and chemokines such as CCL2 and CXCL10. We demonstrate that NF-κB up-regulation is cell-intrinsic and mediated in part by phosphatidylinositol 3-kinase (PI3K) activity induced in response to metabolic activity, which can be moderated by rapamycin treatment. Our observations provide direct evidence that dys-regulated basal NF-κB activity may contribute to the mild pro-inflammatory state of aging.

## INTRODUCTION

Human aging is accompanied by increased serum levels of pro-inflammatory cytokines suggestive of chronic low-grade inflammation. The progressive increase of cytokines such as IL-1, IL-6 and TNFα occurs even in healthy individuals free of cardiovascular diseases and is a risk factor for accelerated decline of physical and cognitive function [1, 2]. Interestingly, age-associated pro-inflammatory state is accompanied by a general decline of immune function that has been termed immunosenescence [3, 4] and results in increased susceptibility of elderly individuals to infectious and autoimmune diseases [5-7], and reduced tumor immune surveillance, which has been hypothesized to contribute to the increasing risk of cancer with aging [8]. The lack of effective response to flu-vaccination in the elderly is a major source of influenza-associated mortality during hospitalization [9, 10].

The basis for age-associated immune dysregulation has been studied primarily in rodent models [11]. One of the most striking features of the aging immune system is a gradual shrinkage of the immune repertoire, with increased proportion of memory cells and reduced proportion of naïve cells in older animals. Because the capability to recognize diverse antigens is a central feature of immune responsiveness, reduction in repertoire is considered to be one of the major determinants of reduced immune efficiency. The imbalance of peripheral lymphocyte homeostasis has also been noted in humans [12]. However, additional cell-intrinsic alterations also occur with age, including impaired cytokine production and reduced proliferation of activated T cells that make it difficult to quantify the relative contributions of reduced repertoire to age-associated immune dysregulation. Studies have shown that the T cell antigen receptor (TCR) itself is functionally impaired with age. One key mechanism in human CD4^+^ T cells is the increased expression of the dual specificity phosphatase DUSP6 from older individuals, which leads to reduced ERK activation in response to TCR Signaling [13]. Such impairment may account for reduced cytokine production and proliferation of cells derived from the elderly. In our studies using human CD4^+^ T cells we found that activation of TCR signaling produces different gene expression in cells obtained from older compared to younger individuals, with a subset of genes including *LRRN3, SERPINB9, ZNF165, LRAP* being under-expressed but also a subset of genes including *IL6, TNIP3, IL1A, LITAF* being over-expressed. Additionally, maintenance of the inducible transcription factor NF-κB was attenuated in CD4^+^ T cells from older individuals.

Age-associated changes in the cellular milieu likely affect the outcome of TCR signaling. Such changes have been studied in both human and mouse T cells. In a study that examined naïve CD4^+^ and CD8^+^ T cells obtained from young and old mice, Mirza et al. found that many genes involved in cell growth, cell death, inflammatory response and cell trafficking were differentially expressed in the old compared to the young mice, both in both pre- and post-TCR stimulated cells. They also found defects in T-cell signaling, cytokine production and Th2 differentiation of post-activated old T cells [14]. A comparison of human CD8^+^ T cells obtained from 5 young (23-27 years) and 4 older (65-80 years) individuals also revealed many genes that were up- or down-regulated between these two age-groups [15]. The cellular pathways most prominently enhanced in the elderly included response to oxidative stress and cytokines, apoptosis, and the MAP kinase kinase (MAPKK) signaling cascade, whereas pathways related to RNA transcription regulation, RNA and DNA metabolism, intracellular transport and the ubiquitin cycle were attenuated [15]. Remodini et al. studied total T lymphocytes in 25 individuals of varying ages and found substantial gene expression changes in the absence of TCR stimulation [16]. A subset of genes whose basal expression increased with age fell into categories that were related to TCR and cytokine signaling. Finally, the most extensive analysis of gene expression changes with age in immune cells was carried out by Ferrucci and colleagues using total peripheral blood mononuclear cells (PBMC). In a large sample size of 733 individuals [17], they found that mRNA splicing was one of the cellular processes that was significantly decreased in older compared to younger participants. Taken together, these studies convincingly demonstrate that immune cells from the aged are altered in many ways, and highlight the importance of understanding mechanisms that underlie these changes. Such mechanistic insight may identify targets for therapeutic intervention to improve immunosurveillance in the elderly.

Here we studied cell-intrinsic differences in human CD4^+^ T cells between individuals of different ages. We demonstrate that in the absence of experimental stimulation gene expression coordinated by the transcription factor NF-κB was up-regulated in human CD4^+^ T lymphocytes from older compared to younger individuals. The mechanism of differential reactivity was cell-intrinsic and modulated by cell metabolic activity. Genes that were up-regulated in T cells from older compared to younger individuals included pro-inflammatory cytokines such as *IL-1* and *IL-6*, and chemokines such as *CCL2* and *CXCL10*. We found that this effect was mediated in part by the activity of phosphatidylinositol 3-kinase (PI3K) and could be moderated by treatment of cells with rapamycin. We propose that reduced ability to maintain metabolic homeostasis in cells from older individuals results in basal up regulation of these inflammatory cytokines, thereby contributing to age-associated chronic inflammation and its consequences on health.

## RESULTS

### Age-dependent alterations in gene expression in rested and unactivated CD4^+^ T cells

**Figure 1:**
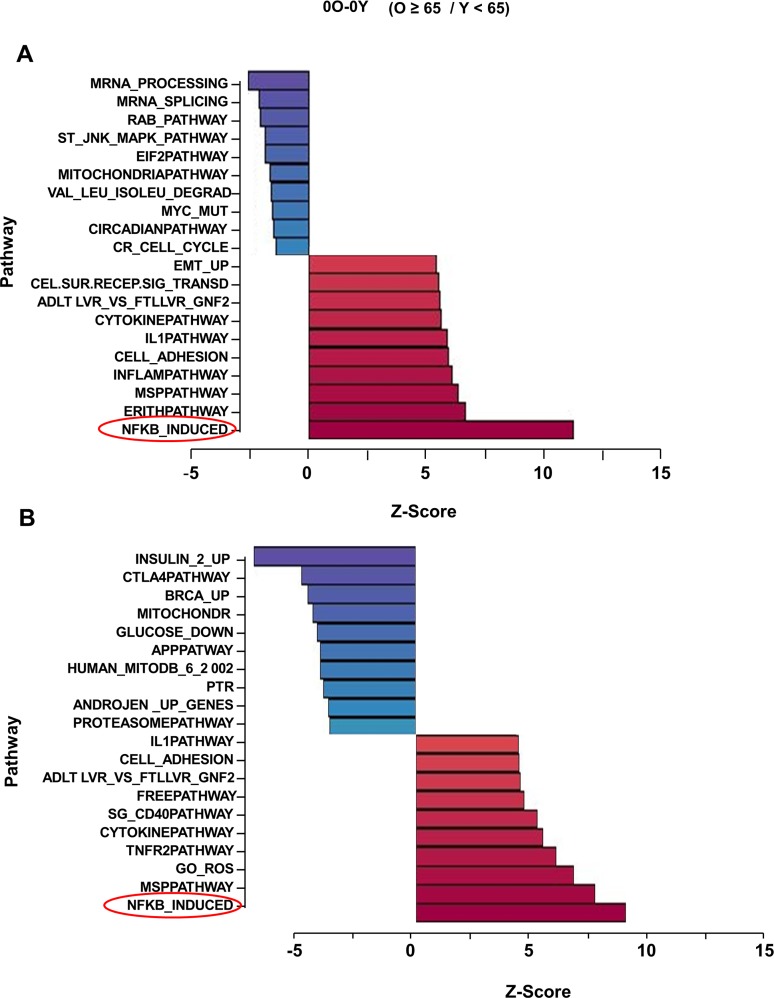
Effect of age on gene expression profiling in unactivated human peripheral blood CD4+ T lymphocytes Total RNA isolated from CD4^+^ T cells that had been incubated for 4h at 37°C was converted to biotin labeled cRNA and hybridized to Illumina HumanRef-8 Expression BeadChip. Normalized hybridization data was analyzed using Parametric Analysis of Gene Set Enrichment (PAGE) (Broad Institute, M.I.T., Cambridge MA). (**A**) a set of 31 donors with age range 25-81 (Table S1A), (**B**) an independent set of 23 donors with age range 26-83 (Table S1B). Pathway Z-scores were averaged amongst less than 65 (Y) and 65 and older (O) individuals and the Z-score difference between O and Y is shown on the X-axis. Each row denotes a different pathway (*p*-value ≤ 0.05 and fdr ≤ 0.3).

To identify changes that accompany immune-dysfunction during human aging, we analyzed gene expression patterns in CD4^+^ T cells from 31 donors ranging in age from 25-81 years (Table S1A). Total cellular RNA isolated from cells incubated at 37°C in the absence of TCR stimulation was converted to biotin labeled cRNA for hybridization to Illumina HumanRef-8 Expression BeadChip. After normalizing signal intensities between samples, we compared average gene expression profiles between participants under 65 years of age (group Y) and those 65 and older (group O). We found 264 differentially regulated genes with absolute value of Z-ratios >1.5 and *p*-value < 0.05 (Table S2). Parametric Analysis of Gene Set Enrichment (PAGE) [18] revealed that the “NF-κB induced” pathway was the most up-regulated in older compared to younger participants (Fig. 1A). Conversely, mRNA processing and splicing pathways were most down regulated in older participants.

NF-κB is an inducible transcription factor that regulates gene expression in cells activated by a variety of stimuli. Gene targets in activated cells include pro-inflammatory cytokines such as *IL1*, *IL6* and *TNFα*. Because high blood levels of the proteins products of these genes are hallmarks of the chronic inflammatory state of aging, NF-κB dysregulation has been implicated in the aging process [19]. However, cells or tissues that are the source of inflammatory markers found elevated in most older individuals have not been identified. Similarly, direct evidence for NF-κB dysregulation at the cellular level is lacking and, consequently, the mechanisms for such dysregulation are unknown. Interestingly, the top 10 up-regulated pathways in our analysis also included “Inflammation”, “Cell adhesion” “IL1” and “Cytokine” pathways, which also contain putative NF-κB target genes, leading us to conclude that cells from older individuals exhibit elevated expression of NF-κB target genes in the absence of overt cell stimulation.

Genes within the “NF-κB induced” pathway that were differentially expressed between cells from younger and older participants are listed in Table S3. These included genes whose protein products are known to induce NF-κB activity such as *IL1β* and *TNFα*, as well as genes that are considered to be targets of NF-κB activity, such as*, TNFAIP2*, chemokines (*CCL2, CXCL2* and *CXCL10*) and cytokines (*IL-lβ, IL6*, and *TNFα*). The set of NF-κB inducers may contribute to the pro-inflammatory state in older individuals, whereas the set of target genes likely contribute to the consequences of age-associated elevation in NF-κB activity. The relatively low statistical significance of individual gene differences in this cross-sectional analysis likely reflected limited sample size in the face of wide heterogeneity due to the high prevalence and variability of clinical and sub-clinical pathology in this population. However, the robust and statistically significant up-regulation of the NF-κB-induced pathway strongly suggests that NF-κB target gene expression increases with age even in resting cells.

To substantiate these observations we replicated this study in an independent group of 23 individuals (Table S1B). By comparing basal gene expression in 18 individuals older than 65 years to basal expression in 5 younger individuals, we found that the “NF-κB-induced” pathway was again the most up-regulated in this set (Fig. 1B). Overall, 6 out of 10 top pathways that distinguished older from younger individuals were shared between the 2 independent experimental groups. These included “IL1”, “Cytokine” and “Cell adhesion” pathways. Additionally, several genes were comparably up-regulated in both data sets, such as *CCL2* and *5*, *CXCL10*, *IL1α* and *β, IL6* and *TNFAIP2* (Table S3), corroborating our hypothesis that these genes may contribute to the age-associated chronic inflammation. Interestingly, we also noticed up-regulation of *NFKB2* and *RelB* genes in both cohorts. These genes are considered to be targets of classical NF-κB (RelA-containing DNA binding activity) [20, 21], but the corresponding gene products comprise components of the non-classical NF-κB pathway that has been implicated in regulating genes that encode chemokines and chemokine receptors [22]. Perhaps age-associated up-regulation of chemokines and their receptors that we observed was a consequence of increased non-classical NF-κB activity in CD4^+^ T cells from older individuals. We conclude that CD4^+^ T cells from older individuals express higher levels of NF-κB target genes.

### Activation of NF-κB-induced pathway is cell-intrinsic

Products of some NF-κB target genes, such as IL-1 and TNF-α, are themselves activators of NF-κB. Thus, NF-κB activation has the potential to establish a self-amplifying feedback loop that may be particularly relevant during aging. It is therefore imperative to distinguish between cause and effect in age-associated NF-κB dysregulation. For example, one explanation for the elevated “NF-κB induced” gene expression signature in the older group could be that these genes were up-regulated as a consequence of higher levels of pro-inflammatory cytokines in the elderly. Alternatively cell-intrinsic elevation of these genes in CD4^+^ T cells from older persons could be the primary and direct cause of chronic inflammatory state associated with aging.

CD4^+^ T cells used in our microarray analyses (Fig. 1) had been incubated at 37°C to serve as controls in our TCR activation studies. We reasoned that elevation of the NF-κB pathway with age noted in these samples could be due to the metabolic activity that occurred during the 37°C incubation (a cell-intrinsic property) or a consequence of the elevated pro-inflammatory environment in the elderly individuals from whom they were obtained. To distinguish between these possibilities we compared gene expression profiles in CD4^+^ T cells that had been held at 4°C during and after purification (to minimize metabolic activity) to the same cells after they had been incubated at 37°C to increase metabolic activity. For this experiment we assayed cells from 23 individuals (age range 29-82) (Table S1C).

**Figure 2:**
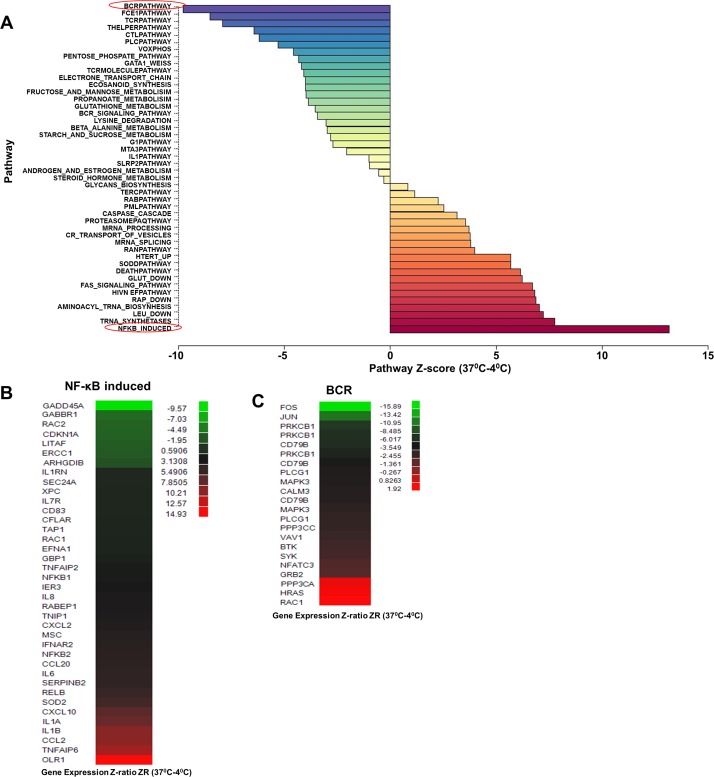
Effect of increased metabolic activity on gene expression profile in CD4+ T lymphocytes RNA samples from 23 donors (age range 29-82) (Table S1C) were obtained from freshly purified CD4^+^ T cells (maintained at 4°C) or from cells incubated at 37°C for 4h. The 46 samples were converted to biotin labeled cRNA and hybridized to Illumina HumanRef-8 Expression BeadChip. (**A**) Normalized data were analyzed by PAGE and the average Z-score for individual pathways compared between 4°C samples and those incubated at 37°C to obtain a Z-score difference x-axis; Z-score (37°C-4°C ). Rows denote individual pathways. Most prominently up- and down-regulated pathways at 37°C are indicated in red. (**B**) and (**C**) Statistically significant genes within the NF-κB induce (up-regulated) and BCR (down-regulated) pathways.

We found that the “NF-κB-induced” pathway was prominently induced in cells from both younger and older individuals (Fig. S1A,B) that had been incubated at 37°C compared to those maintained at 4°C (Fig. 2A). The difference between Y and O (Fig. 1) was therefore due to the increased activation of “NF-κB-induced” pathway in CD4^+^ cells from older individuals. Age associated changes in NF-κB-induced pathway were not evident in cells maintained at 4°C. Genes within the pathway whose expression increased with 37°C incubation included *OLR1, IL-1α, β* and *IL-6*, and the chemokines *CCL2* and *CXCL10* (Fig. 2B, Fig. S1C). We confirmed the microarray results by quantitative RT-PCR analysis using RNA from three subjects for a subset of these genes (Fig. S1D). These observations suggest that metabolic activity up-regulates NF-κB function in a cell-intrinsic manner, resulting in the predisposition of CD4^+^ cells from older individuals to transcribe pro-inflammatory genes.

The pathway that was most down-regulated by *ex-vivo* incubation was the “BCR pathway (Fig. 2A),” within which significantly changed genes included immediate early response genes such as *FOS* and *JUN*, and components of BCR signaling such as *CD79B* and *PLCγ1* (Fig. 2C). Our interpretation is that activity of the “BCR pathway” in cells maintained at 4°C reflected low-grade stimulation via TCR/MHC interactions *in vivo* [23]. To the best of our knowledge, this is the first demonstration of the direct gene regulatory consequence of this form of “tonic” TCR signaling. Absence of MHC ligands during the 37°C incubation of purified CD4 T cells down-regulated this pathway and replaced it with other, non-TCR dependent consequences of metabolic activity. One of the most important consequences was activation of a subset of NF-κB-dependent genes.

To further strengthen the idea that the effects of *ex vivo* incubation were due to cell-intrinsic characteristics, we carried out the same experiments in serum-free medium. For these studies CD4^+^ T cells from 8 donors (age range 30-75) (Table S1D) were placed immediately after purification in normal medium (containing fetal bovine serum) or serum-free medium. Thereafter, the cells were either maintained at 4°C or incubated for 4h at 37°C, followed by RNA analysis. We found that 15 out of 20 top pathways up-regulated by 37°C incubation, including “NF-κB-induced”, were the same between cells incubated in normal or serum-free medium (Fig. S2A). That several of the shared pathways were related to cellular metabolism (such as “Rap”, “Glut” and “Leu down”, “Trna synthetases”, “Trna biosynthesis”), was consistent with our starting hypothesis that the effects of cell incubation at 37°C were due to metabolic activation. We propose that up-regulation of the “NF-κB induced” pathway in cells incubated at 37°C is a consequence of cellular metabolism.

Interestingly, consequences of incubation in normal medium and serum-free medium were similar but not identical. For example, the order of the top 20 most-induced pathways differed between the two conditions, with the “NF-κB-induced” pathway changing from being the most highly up-regulated in normal medium to the middle of the list in serum-free conditions. To get a molecular measure of differences between normal and serum-free medium, we compared genes within the “NF-κB-induced” pathway that were induced by 37°C incubation. The top 10 genes induced in serum-free medium were also induced in normal medium, though many more genes were induced in normal medium (Fig. S2B). In the absence of further analysis, we will consider only the shared genes as reflecting the effects of metabolic activity.

### Involvement of PI3K in metabolism-induced gene activation

Increased reactive oxygen species (ROS) produced by metabolic activity is an obvious candidate for activating the “NF-κB-induced” pathway in cells incubated at 37°C [24]. One of the genes found to be maximally induced by 37°C incubation was *OLR1* (also known as *LOX1*), a gene that has been previously implicated in oxidative stress signaling [25, 26]. However, we found that mitochondrial ROS generated by antimycin treatment of CD4^+^ T cells (Fig. S3A) did not increase *OLR1* or *TNFAIP6* expression (Fig. S3B). Conversely, incubation of CD4^+^ cells with N-acetylcysteine, a well-characterized ROS quencher, failed to suppress *OLR1* activation (Fig. S3B). Based on these observations, we ruled out mitochondrial ROS to be a major inducer of NF-κB target genes under these conditions.

**Figure 3:**
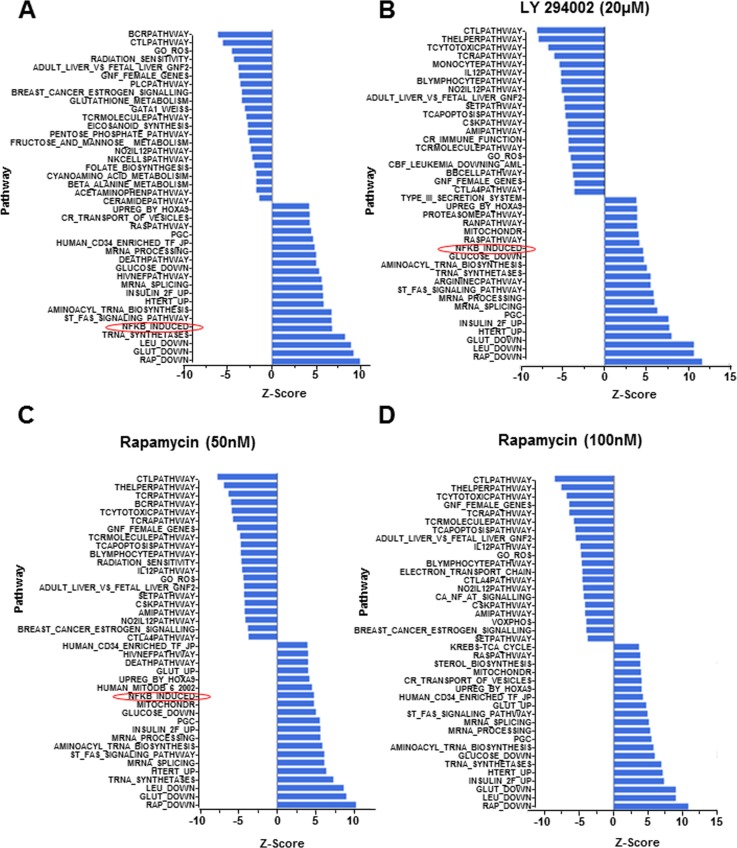
Gene expression following pharmacologic perturbation of PI3K in CD4+ T lymphocytes CD4^+^ T cells were incubated at 37°C for 4h in the absence of pharmacologic agents or in the presence of LY 294002 (20μM) or rapamycin (50nM or 100nM, as indicated). RNA isolated from freshly purified cells or cells treated as above was converted to biotin labelled cRNA and hybridized to Illumina HumanRef-8 Expression BeadChip. Normalized data were analyzed by PAGE and the average Z-score for individual pathways compared between fresh samples (maintained at 4°C and (**A**) cells incubated in the absence of inhibitors, (**B**) cells incubated in the presence of 20μM LY 294002, (**C**) cells incubated in the presence of 50nM rapamycin and (**D**) cells incubated in the presence of 100 nM rapamycin. Rows denote individual pathways.

The phosphatidylinositol 3-kinase (PI3K) pathway has long been implicated in aging based on the demonstration that attenuating insulin/IGF1/FOXO activity extends lifespan in *C. elegans* [27]. Moreover mutation in the *age-1* gene, which encodes the catalytic subunit of PI3K, also extends longevity in *C. elegans* [28]. The relevance of this axis for aging has been confirmed in rodents [29], and there is evidence that it is a key mediator of caloric restriction [30], the only known universal extender of life/health span across species. Accordingly, the immune suppressant rapamycin, that affects a subset of responses mediated by PI3K, is the only treatment demonstrated to prolong longevity in mammals [31, 32]. Despite substantial information in model organisms, little is known about PI3K dysregulation during human aging. To test whether exacerbation of “NF-κB-induced” pathway in CD4^+^ T cells from older individuals involved the PI3K pathway we assayed the effects of culturing CD4^+^ T cells in the presence of the PI3K inhibitor LY 294002, or rapamycin. RNA purified from these cells was used for microarray analysis. We found that 9 out of the top10 pathways up-regulated by 37°C incubation were unaffected by either drug (Fig. 3). In contrast, activation of the “NF-κB-induced pathway was substantially inhibited by pre-treatment with both LY 294002 and with rapamycin (Fig. 3B-D).

**Figure 4:**
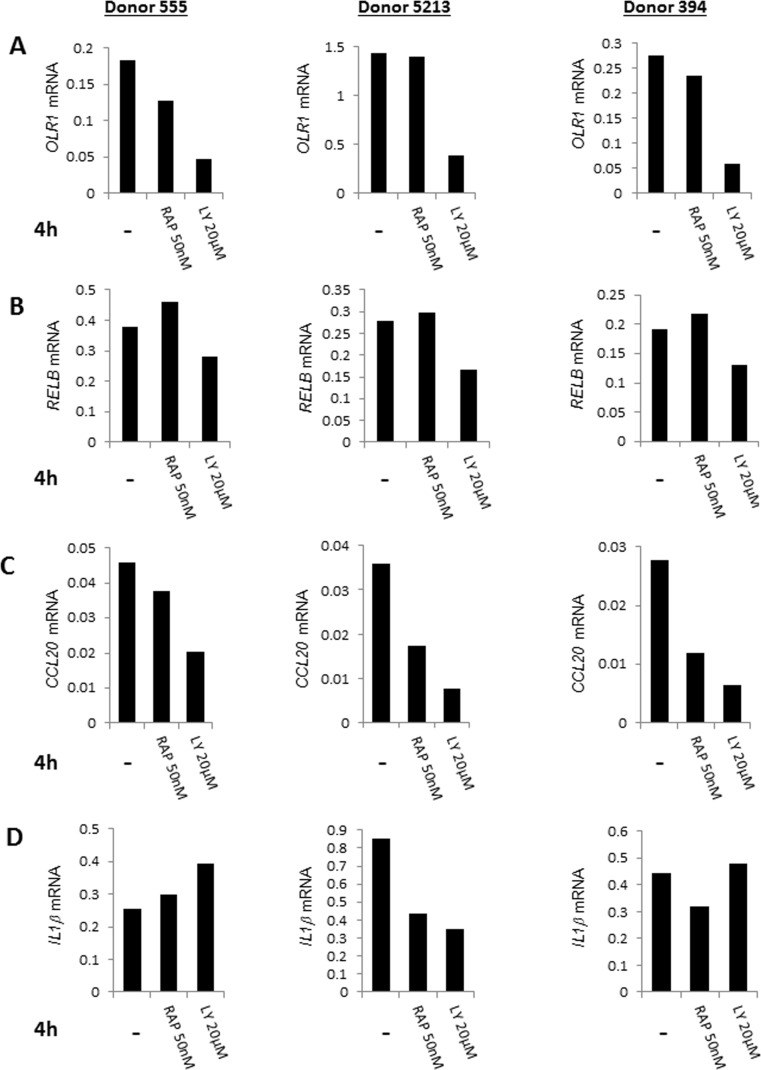
Quantative RT-PCR validation of the effects of pharmacologic agents on gene expression in CD4+ T lymphocytes CD4^+^ T cells from three donors (ages 30, 63, 73) were cultured at 37°C for 4h in the presence of LY294002 (20μM), rapamycin (50nM) or in the absence of added drugs. RNA prepared from these cells was assayed for expression of (**A**) *OLR1*, (**B**) *RELB*, (**C**) *CCL20* and (**D**) *IL1β* by quantitative RT-PCR. Expression values after normalization to GAPDH are shown on the Y axis.

We validated these observations by quantitative-RT-PCR analysis using RNA from three additional individuals. *OLR1*, *RELB and CCL20* gene activation was consistently reduced in the presence of LY 294002 in CD4^+^ T cells from three independent donors (Fig. 4A-C) (Fig. S4A-D). We conclude that PI3K affects cell-intrinsic induction of NF-κB target genes. The observed variability and the incomplete suppression (e.g. of *RELB*) was entirely consistent with the more global gene expression analysis that indicated that activation of the NF-κB-induced pathway was not completely abrogated by either drug. An additional example of variability was demonstrated by analysis of *IL-1β* gene induction whose expression was suppressed in only one out of the three subjects. Unlike the observed effects with LY 294002, specific NF-κB target genes that we analyzed by quantitative RT-PCR were not suppressed by rapamycin. This is consistent with the greater involvement of mammalian target of rapamycin (mTOR) with protein translation rather than transcription [33]. We conclude that age-associated elevation of NF-κB target gene expression as a consequence of cell-intrinsic metabolic activity is mediated in part by PI3K.

## DISCUSSION

We demonstrate that putative NF-κB target genes are up-regulated in the absence of overt cell stimulation in CD4^+^ T cells from older compared to younger persons. This observation was validated in two-independent cohorts comprising of 31 and 23 individuals. Genes within this pathway that were reproducibly up-regulated in both cohorts included cytokines and chemokines such as *IL-1α, β, IL-6, IL-8, CCL2, CCL5, CXCL10* and *TNF*, the inflammatory markers *TNFA1P2* and *6*, and genes encoding the redox-responsive enzymes *SOD2*. Additionally, we provide evidence that this property is cell intrinsic. Because our *ex vivo* experiments showed that CD4^+^ T cells from all age groups up-regulated the “NF-κB-induced” pathway in response to increased metabolic activity, we interpret the differences noted between older and younger cohorts as indicating that cells from older individuals were more prone to activate expression of NF-κB target genes. We propose that this increased propensity may, in part, underlie the chronic inflammatory phenotype found in most older persons.

Based on our current experiments, we cannot distinguish whether PI3K/NF-κB up-regulation occurs at a low level in the majority of cells or in a fraction of cells. Our working hypothesis is that the phenotype we observed results from a small fraction of cells where PI3K activation exceeds a threshold. Continued activation of NF-κB target genes even in a subset of cells may well contribute to the gradual accumulation of age-associated inflammatory cytokines in serum.

In searching for mechanisms by which metabolic activity induced NF-κB target gene expression, we identified a role for PI3K in this process but not for mitochondrial ROS. The latter observation was surprising since mitochondrial “dysfunction” is considered to contribute prominently to the aging process [34] and NF-κB is known to be a ROS-responsive transcription factor. The involvement of PI3K was interesting from the NF-κB perspective as well as from the aging perspective. While all NF-κB-inducing stimuli do not involve PI3K, studies in both B and T lymphocytes show that PI3K is required for NF-κB induction in response to antigen receptor stimulation [13]. In our experiments, metabolic activation was clearly not initiated at the antigen receptor. Indeed, the “BCR-pathway” was the one that was maximally down-regulated in CD4^+^ T cells incubated *ex vivo*. Though the initiating stimulus remains unknown, the requirement for PI3K will help identify other possible components in this age-associated signal transducing pathway that activates expression of NF-κB target genes. Alternatively, some studies indicate that Akt, a kinase activated by PI3K, can directly induce NF-κB. It remains to be determined whether NF-κB activation in response to metabolic activity requires Akt and this hypothesis should be addressed in other studies.

PI3K has featured prominently in lower organisms as a mediator of aging. In classic studies in *C. elegans*, loss of the PI3K responsive transcription factor DAF16 was shown to increase life span [35, 36]. In worms DAF16 lies at the end of a signal transduction cascade initiated by insulin-like growth factor (IGF1). The forkhead family of transcription factors (of which DAF16 is the *C. elegans* ortholog) have since been shown to control longevity in *Drosophila melanogaster* as well [37]. A direct role for Forkhead proteins in mammalian aging has been more difficult to ascertain because there are multiple family members that can provide compensatory function in the absence of a subset of factors. Nevertheless, the longevity phenotype of growth hormone and IGF-1-deficient mice [38, 39] and, more recently, the anti-aging effects of systemic administration of rapamycin in mice [32, 40] is strongly consistent with a role for PI3K during mammalian aging.

To the best of our knowledge, the precocious PI3K activation in CD4^+^ T cells from older individuals presented here is the first direct demonstration of cellular dysregulation of this pathway during human aging. We propose that PI3K activates a subset of pro-inflammatory cytokines and chemokines, in particular, those that respond to the transcription factor NF-κB. These observations are consistent with the recently proposed hyper-function theory of aging which posits that inappropriate PI3K activation drives many aging phenotypes via mTOR activity [41, 42]. Indeed, inhibition of mTOR in *in vitro* models of cellular senescence has been shown to to suppress a senescent phenotype [43, 44]. In the case of primary human CD4^+^ T cells presented here, we found that rapamycin treatment ameliorated aging-associated gene expression patterns, demonstrating that the mTOR pathway was up-regulated in cells from older individuals. However, inhibition of PI3K had greater effects than rapamycin treatment, suggesting that PI3K hyper-activity contributes more than mTOR activation towards the full aging phenotype. The Akt/FoxO pathway could be one such additional pathway that cooperates with mTOR to manifest the complete aging program. Our observations highlight the generality of this aging mechanism and its potential for control by drugs such as rapamycin.

Within the NF-κB-induced pathway we also noted that genes encoding *NFKB2* and *RELB* were up-regulated in both groups that we studied. These proteins constitute the alternate NF-κB response that is initiated at receptors that bind lymphotoxin and B cell activating factor (BAFF) [45]. Whereas the classical NF-κB pathway drives expression of pro-inflammatory genes such as *TNFα, IL-1, IL-6* and *IFN-γ*, the alternate NF-κB response is skewed towards chemokines and their receptors [22]. This may be the source of increased chemokine gene expression that we noted in CD4^+^ T cells from older individuals. Increase of both classical and alternate NF-κB pathways may therefore underlie many of the chronic inflammatory symptoms associated with aging.

Despite consistent demonstration of age-associated inflammation in gene expression studies in humans [46-48] and the substantial evidence for the role of NF-κB in mouse models, the two have rarely been experimentally connected in the context of human aging. Indeed, the most substantive indication of a connection of NF-κB to human aging appears to be the prevalence of NF-κB target cytokines, such as IL-6, TNFα and IL-1, in serum. Our observations provide the first cellular demonstration of NF-κB dysregulation leading to cytokine and chemokine gene expression during human aging. Moreover, the identified involvement of PI3K provides a plausible mechanism for NF-κB activation and a shared mechanism of aging that is active from invertebrates to mammals. We do not intend to imply that CD4^+^ T cells studied here are the only, or even the major, source of age-associate cytokines. Rather, we propose that our studies exemplify the principle that dysregulated PI3K activity can, via NF-κB, contribute to chronic inflammation associated with human aging.

## METHODS

### Subjects

Thirty one donors (17 men and 14 women; Table S1A), age range 25-81 years old, were enrolled as part of the original cohort (group 1) and 23 donors (18 men and 5 women; 1B), age range 26-77 years old, were enrolled for purposes of replication (group 2). In addition, 23 donors, age range 29 – 82 years old, from a combination of participants from group 1, 2 or additional enrollees were studied for temperature response of their CD4^+^ T cells at 4°C and 37°C. Also eight extra donors, age range 30-75 years old, were tested for temperature response of CD4^+^ T cells using RPMI and serum-free medium. All blood samples were obtained after informed consent of the individuals in the study. The subjects for this study were selected from the Baltimore Longitudinal Study on Aging (BLSA).

As previously described [49] BLSA participants are volunteers that at the time of study enrollment are “healthy” based on very strict eligibility criteria, which include no major chronic diseases (such as coronary heart disease, congestive heart failure, peripheral artery disease, cancer, chronic pulmonary diseases, diabetes, severe osteoarthritis and others), no chronic drug treatment except low dose Aspirin, antihypertensive drugs and statins, no mobility impairment and or disability (ability to walk 400 meters without stopping and without developing symptoms), no cognitive impairment (Mini Mental State Examination score >26 and less than 3 errors in the Blessed Mental Status), no joint replacement, no osteoporotic fractures. Thus, BLSA participants tend to be healthier than the general population. In addition, this study required a large quantity of WBC that could only be obtained by cytapheresis. Eligibility for cytapheresis in the BLSA is based on even more strict criteria based on the American Association for Blood Bank Criteria for whole blood donation. Thus, participants in this study were exceptionally healthy, and therefore, the difference between the two groups should be attributable to aging “per se”. BLSA has continuing approval from the Institutional Review Board of the MedStar Research Institute. Participants provided informed consent for all analyses included in this study.

### Cell preparation

Peripheral blood mononuclear cells (PBMC) were isolated from heparinized blood using Ficoll-Paque Plus (GE Healthcare, Piscataway, NJ, USA) density gradient centrifugation as described previously [49]. CD4^+^ T cells were obtained by positive selection using anti-human CD4 microbeads (Miltenyi Biotec Auburn, CA, USA).

### CD4^+^ T cell

For gene expression studies 1×10^7^ CD4^+^ T cells in 3 ml RPMI 1640 medium were incubated for 4h at 37°C (5% CO_2_ incubator). To test the effects of metabolic activity two lots of 1×10^7^ CD4^+^ T cells in 3 ml RPMI 1640 medium were prepared. One was incubated for 4h at 37°C (5% CO_2_ incubator), the other was left at 4°C. All cells were held before incubation at 4°C. To test the effects of serum 5×10^6^ CD4^+^ T cells in 3 ml of RPMI 1640 or AIM V (Life Technologies, Carlsbad, CA, USA) serum-free medium were held at 4°C or incubated for 4h at 37°C (5% CO_2_ incubator). After incubation cells were harvested, washed once with cold PBS and collected by centrifugation. Total cellular RNA was prepared by lysing cell pellet in 350μl RLT buffer according to the manufacturer's recommenda-tions (QIAGEN Inc, Valencia, CA, USA).

### Flow cytometry with MitoSOX

1×10^6^ CD4^+^ T cells were treated with 5mM N-acetyl-cysteine (Sigma-Aldrich, St Louis, MO, USA) in 1ml RPMI 1640 medium for 4h at 37°C (5% CO_2_ incubator). Also, 1×10^6^ cells were exposed to no ROS treatment in either RPMI medium or in phosphate buffered saline (PBS) without calcium and magnesium (Quality Biological, Gaithersburg, MD, USA) in similar conditions. In addition, duplicate plates were incubated at 4°C. MitoSOX Red mitochondrial superoxide indicator (Invitrogen, Carlsbad, CA, USA) was added to all the dishes to a final concentration of 5μM according to the manufacturer's recommendations during the last 30 minutes at 37° C. Cells were then washed twice with warm PBS without calcium and magnesium. Positive control dishes cells were treated with 50 μM antimycin A (Sigma-Aldrich) for additional 30 min at 37° C. Mitochondrial superoxide was measured by flow cytometry using Canto II FACS (BD Bioscience, Franklin lakes, NJ, USA).

### Drug treatment of CD4^+^ T cells

5×10^6^ CD4^+^ T cells were plated in 6 well plates in RPMI medium. CD4^+^ T cells were treated with varying concentrations of rapamycin (Calbiochem-EMD MILIPORE Darmstadt, Germany; 25nM, 50nM, 100nM, 200nM, 500nM and 1000nm respectively) and also LY 294002 (Calbiochem-EMD MILIPORE; 5μM, 10μM, 20μM, 25μM, 30μM, 40μM). Control wells were also created without any added drug. Plates were then incubated for 4 h in 5% CO_2_ at both 37°C and 4°C.

### Total RNA purification and Real time PCR

Total RNA was extracted from frozen cell pellets (5×10^6^ cells) using the Qiagen RNeasy Mini Kit (QIAGEN Inc). cDNA was synthesized using random hexamers and the SuperScript First Strand Synthesis System ( Invitrogen Life Technologies). Real time PCR was performed in duplicates using the AB 7500 Real Time PCR System (Applied Biosystems, Foster City, CA, USA). Expression of *OLR1, RELB, CCL20, IL1β*and *TNFAIP6* were normalized to *GAPDH* mRNA on the same PCR plate. Prior to microarray analysis, the RNA quality and quantity were checked using an Agilent 2100 bio-analyzer and RNA nano chips. Primer sequences used for PCR are shown in Table S4.

### Microarray hybridizations

Total RNA was used to generate biotin-labeled single-strand RNA (cRNA) as previously described [49] using the Illumina TotalPrep RNA Amplification Kit (Ambion, Austin, TX) according to the manufacturer's recommendation. A total of 0.75μg of biotin-labeled cRNA was hybridized at 58°C for 16 hours to Illumina's Sentrix HumanRef-8 Expression BeadChips (Illumina, San Diego, CA, USA). The arrays were washed, blocked and the labeled cRNA was detected by staining with streptavidin-Cy3. The arrays were scanned using an Illumina BeadStation 500X Genetic Analysis Systems scanner and the image data extracted using Illumina BeadStudio software, version 3.0.

### Microarray data analysis

Microarray data was analyzed as previously described [49] using DIANE 6.0. Raw microarray data were subjected to filtering by the detection *p*-values than normalized by Z-transform with log signal values; the data are further tested for significant changes as previously described. Individual genes with Z-ratio ≥ 1.5, *p*-value ≤ 0.05 and fdr ≤ 0.3 were considered significantly changed. The Parametric Analysis of Gene Enrichment (PAGE) algorithm was employed for gene set enrichment analysis by using all of the genes in each sample as input against and the data set supplied by Gene Ontology Institute and pathway gene set of MIT Broad Institute molecular signature database. For each relevant comparison, the lists of differentially expressed genes and Z-ratios were entered into the PAGE Pathway Analysis software to organize them according to known biological pathways. The enrichment Z-scores for each functional grouping were calculated based on the chance of mRNA abundance changes predicting these interactions and networks by Z-test. Pathways *p*-value ≤ 0.05 and pathways fdr ≤ 0.3 are the cutoff criteria for the significant pathway selection.

### Accession Numbers

The microarray GEO accession numbers for the data reported in this paper is GSE62376. This is the super series that includes all 5 experiments, each with their own numbers (links are not released).

## SUPPLEMENTAL DATA FIGURES AND TABLES


