# Supplementary material for: Age-associated changes in basal NF-κB function in human CD4+ T lymphocytes via dysregulation of PI3 kinase
Source: Aging (Albany NY). 2014 Dec 6;6(11):957–69. doi: 10.18632/aging.100705 (PMC4276789; doi:10.18632/aging.100705)
Supplement: Supplementary file 1 [file aging-06-957-s001.pdf]

Figure S1A, B, C

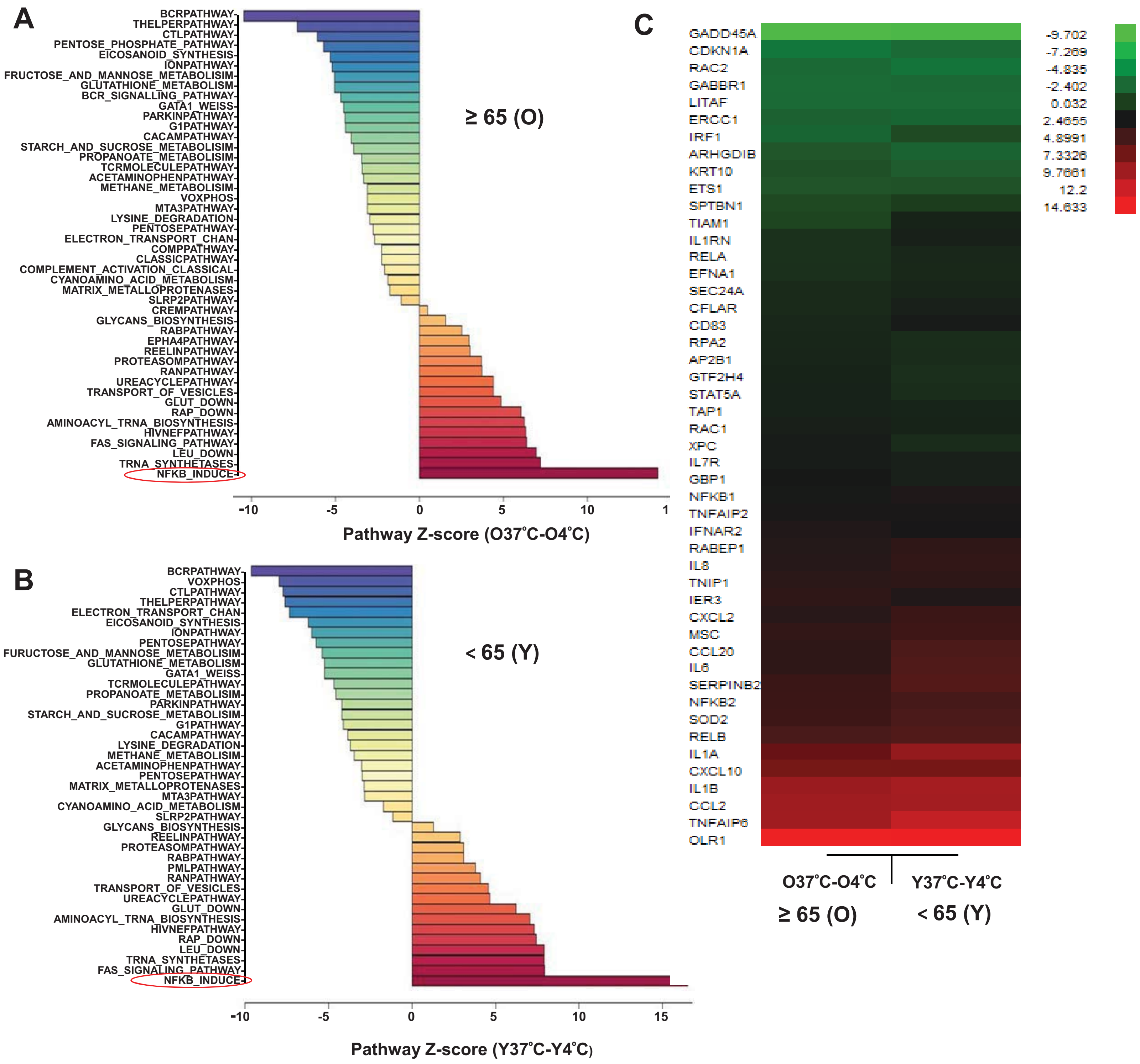

Figure S1D

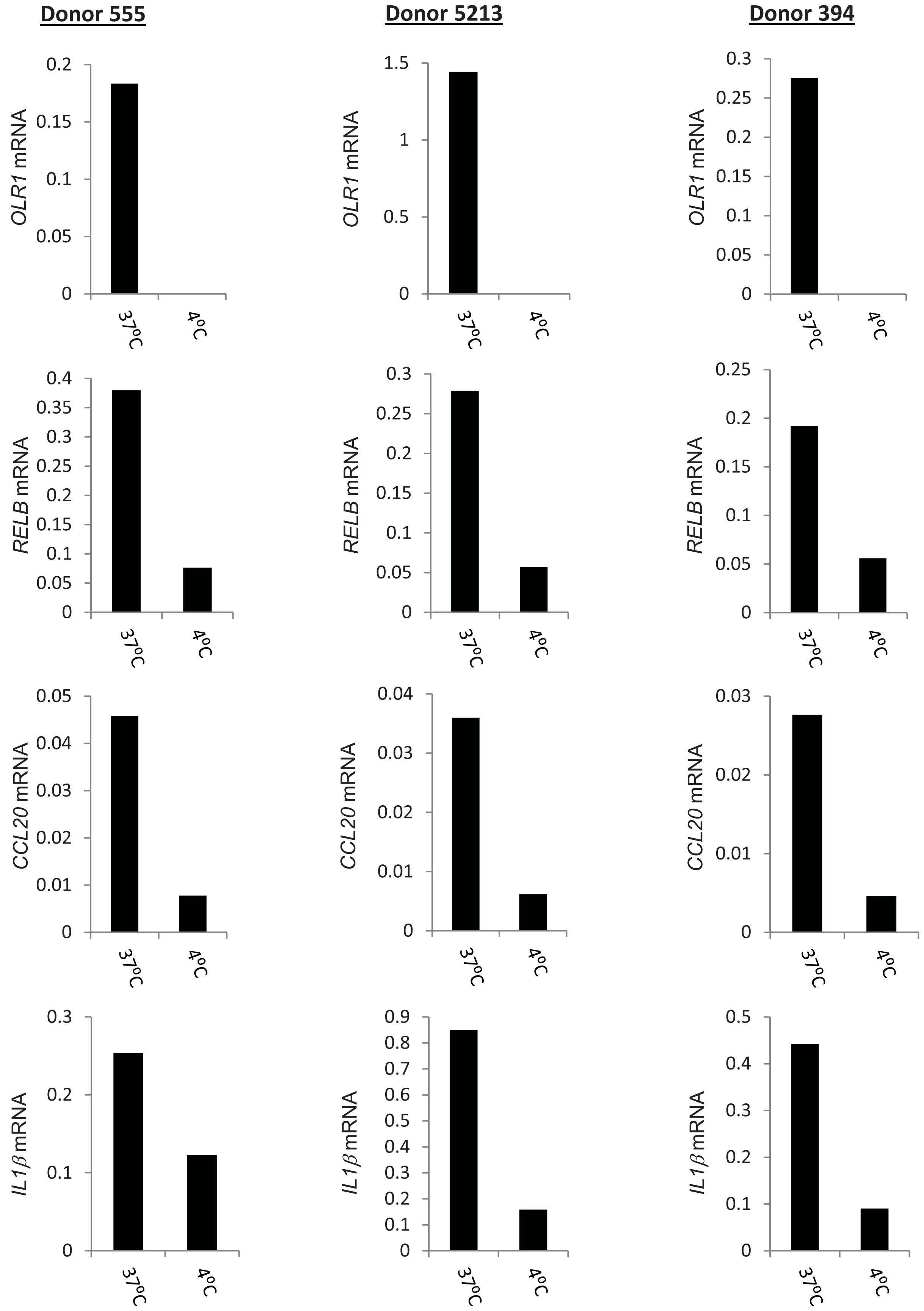
Donor 555
Donor 5213
Donor 394
OLR1 mRNA
RELB mRNA
CCL20 mRNA
IL1β mRNA
37°C
4°C

Figure S1. Gene expression changes in $CD4^+$ T lymphocytes cultured at 37°C for 4h. $10x10^6$ $CD4^+$ T cells from 23 individuals (age range 29-82) (Table S1C) were either kept at 4°C during and after purification, or cultured at 37°C for 4h. Total RNA prepared from these cells was analyzed by microarrays as described in the Methods section. Average gene expression profiles were compared between samples maintained at 4°C to those incubated at 37°C. Pathways maximally up- or down-regulated by 37°C incubation were determined by Parametric Analysis of Gene Set Enrichment (PAGE, Kim and Volsky 2005) in A) subjects aged 65 years or older (n=8) and B) subjects younger than 65 years (n=15). C) Genes within the NF-κB-induced pathway whose expression was significantly changed (*p*-value ≤ 0.05, Z-ratio ≥ 1.5) by *ex vivo* culture. Averaged data from subjects ≥ 65 yrs and subjects less than 65 years are shown in the left and right columns, respectively. D) $CD4^+$ T cells from three individuals were cultured at 37°C or maintained at 4°C. RNA from these cells were assayed by quantitative RT-PCR for the indicated genes using the AB 7500 Real Time PCR System (Applied Biosystems). Primer sequences are shown in Table S4. Expression values after normalization to GAPDH are shown on the Y axis.

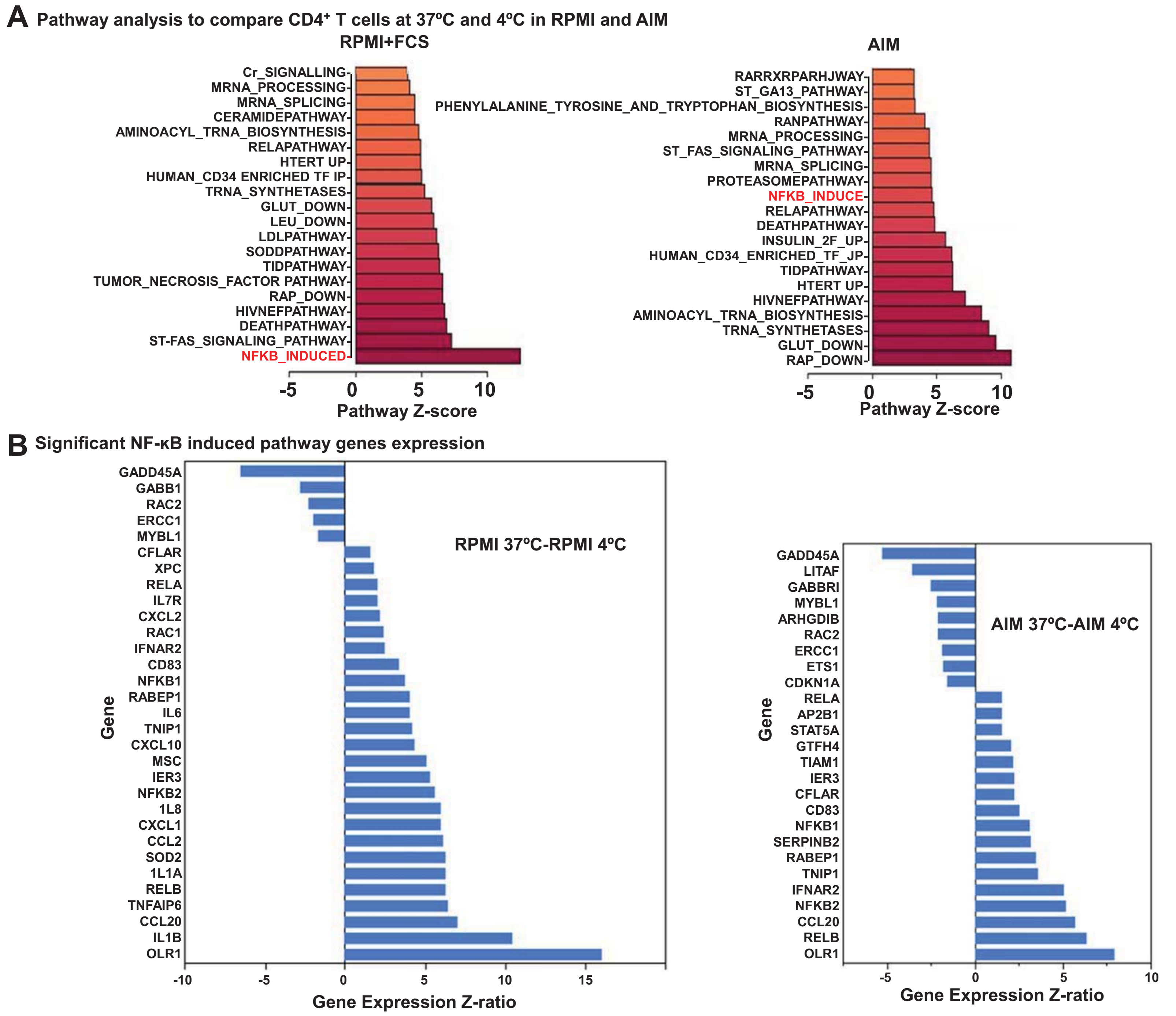

Figure S2 Gene expression analysis in CD4+ T Cells cultured in different media. CD4+ T cells from 8 individuals (age range 30-75) (Table S1D) were cultured in RPMI medium containing 10% fetal calf serum (labeled RPMI) or synthetic medium (labeled AIM, catalog # 12055091 Life Technologies) lacking FCS for 4h at 37°C. Total RNA prepared from these cells was analyzed by microarrays as described in the Methods section. Average gene expression profiles were compared between cells cultured in either medium to cells maintained at 4°C. A) Up-regulated pathways determined by PAGE in RPMI medium (left panel) or AIM (right panel) are shown. B) Genes within the NF-κB-induced pathway whose expression was significantly changed (*p*-value ≤ 0.05, Z-ratio ≥ 1.5) in either culture condition.

Figure S3

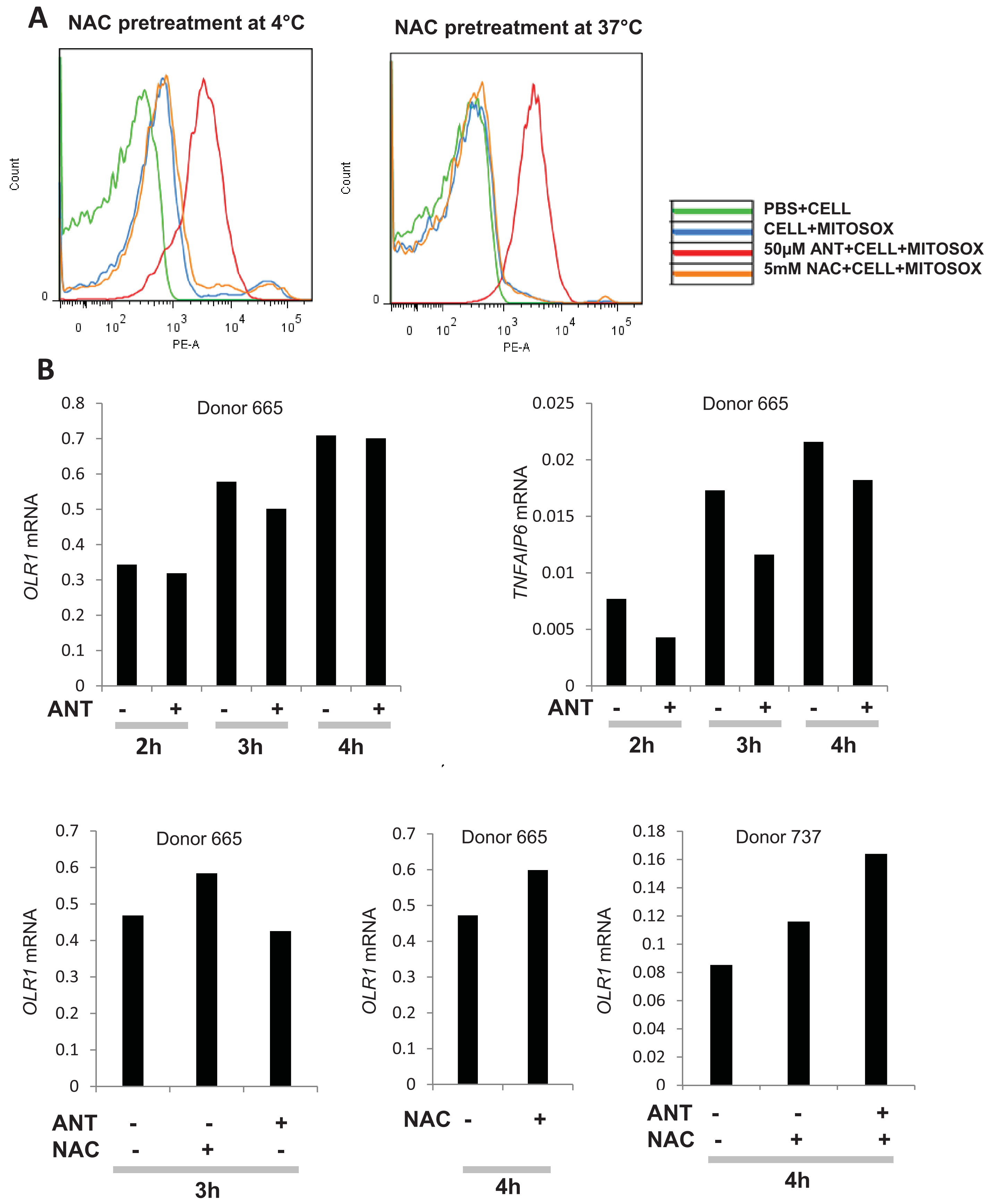
A
NAC pretreatment at 4°C
NAC pretreatment at 37°C
Count
PE-A
PBS+CELL
CELL+MITOSOX
50µM ANT+CELL+MITOSOX
5mM NAC+CELL+MITOSOX
B
Donor 665
OLR1 mRNA
TNFAIP6 mRNA
ANT
NAC
2h
3h
4h
Donor 737

Figure S3. Role of mitochondrial oxidative stress in regulating gene expression program in CD4$^+$ T cells cultured *ex vivo*. A) Representative flow cytometric profiles of CD4$^+$ T cells incubated at 37°C for 4h. Cells were treated with 50 µM antimycin A (Ant) to induce mitochondrial ROS, 5mM N-acetyl cysteine (NAC) to quench cellular ROS or left untreated (PBS and mitosox only) for the duration of culture. Cells were labeled with MitoSOX Red mitochondrial superoxide indicator (5µM, Invitrogen) according to the manufacturer's recommendation and analyzed using a BD FACS Canto II cytometer. B) Quantitative RT-PCR analysis of gene expression in cells treated as indicated. Experiments were carried out with cells from two donors. Test gene expression was normalized to GAPDH for each comparison.

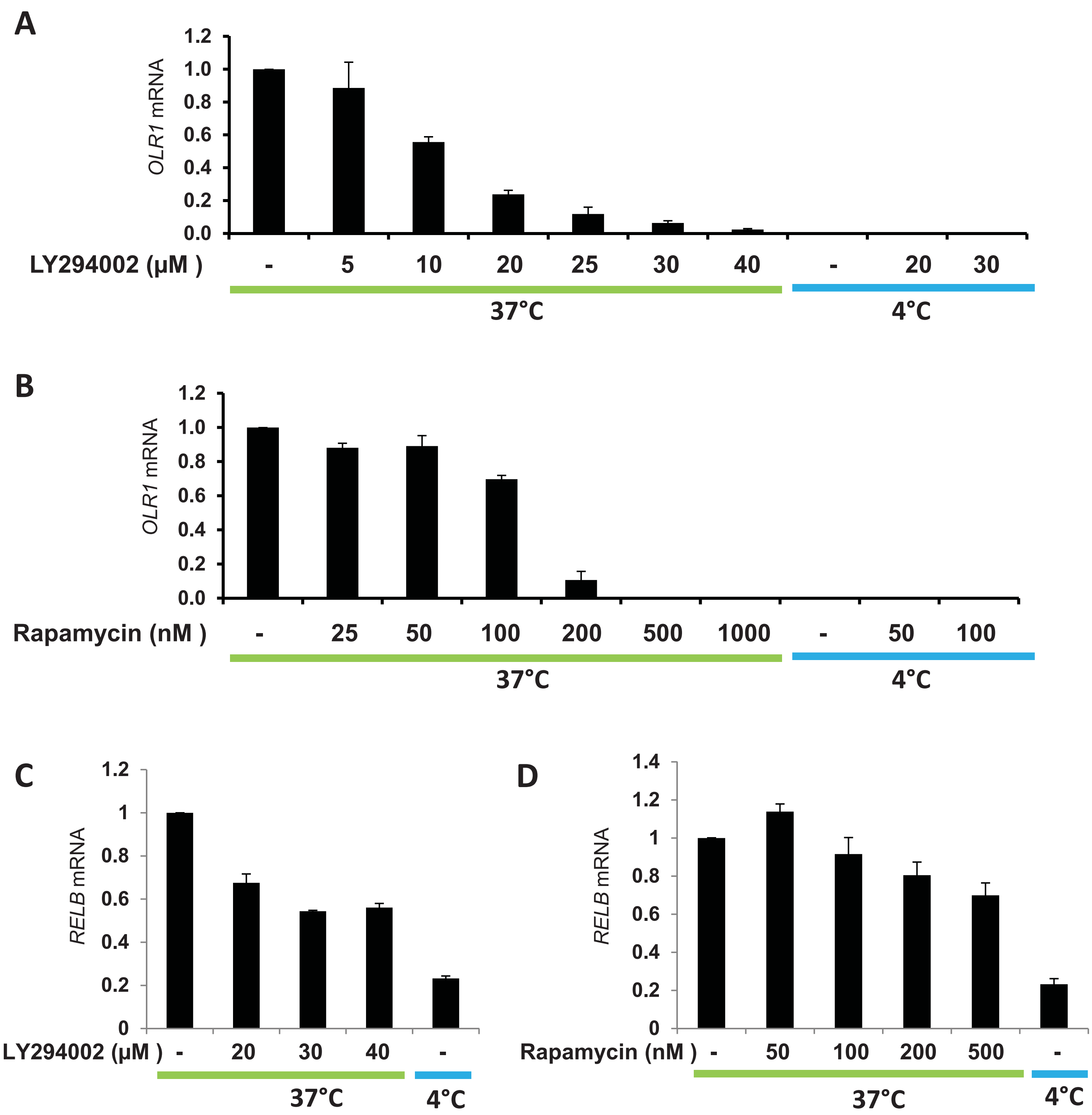

Figure S4. Effect of LY294002 (PI3 kinase inhibitor) and rapamycin (mTOR inhibitor) on gene expression induced by *ex vivo* culture of $CD4^+$ T Cells. RNA isolated from $CD4^+$ T cells that were kept at 4°C or incubated at 37°C in RPMI medium for 4h in the presence of various concentrations of LY294002 or rapamycin as indicated was analyzed by quantitative RT-PCR for expression of *OLR1* or *RELB* genes. The dose range of LY294002 and rapamycin did not reduce cell viability over the time course of the experiment. Error bars reflect the standard error of the mean (±SEM**).** Test gene expression was normalized to GAPDH for each comparison.

Table S1A. Group 1 Donors' Sex and Age

Donor #	Sex	Age	
ND29	M	25	
ND60	F	29	
ND33	M	30	
BL10	M	30	
ND55	M	40	
ND57	F	40	
ND58	F	41	
ND53	M	43	
ND32	M	52	
ND36	M	52	<65
ND59	M	52	
BL01	F	55	
BL08	F	55	
BL21	M	55	
BL11	F	57	
BL04	M	60	
BL12	M	60	
BL19	F	61	
BL20	F	62	
BL18	M	63	
BL03	F	68	
BL16	F	70	
BL17	M	70	
ND31	F	71	
BL06	F	71	
BL22	M	72	≥65
BL07	M	73	
BL09	M	75	
BL13	F	75	
BL05	M	77	
BL02	F	81	

Table S1B. Group 2 Donors' Sex and Age

Donor #	Sex	Age	
ND703	M	26	
ND691	M	28	
ND340	M	30	<35
ND554	M	33	
BL60	F	35	
BL75	F	66	
BL61	M	66	
BL52	F	68	
BL73	F	68	
BL57	M	70	
BL76	M	71	
BL56	M	72	≥65
BL58	M	73	
BL67	M	73	
BL68	M	73	
BL70	M	74	
BL63	M	75	
BL59	M	75	
BL78	F	76	
BL66	M	76	
BL49	M	77	
BL64	M	83	
BL74	M	83	

Table S1C. Donors' Sex and Age
(CD4+ T cells were incubated for 4h at 37°C and 4°C)

Donor #	Sex	Age	Degree
ND60	F	29	4
ND60	F	29	37
BL10	M	30	4
BL10	M	30	37
BL60	F	35	4
BL60	F	35	37
ND55	M	40	4
ND55	M	40	37
BL25	M	44	4
BL25	M	44	37
BL35	F	49	4
BL35	F	49	37
BL44	M	44	4
BL44	M	44	37
BL27	M	52	4
BL27	M	52	37
BL28	M	56	4
BL28	M	56	37
BL33	M	54	4
BL33	M	54	37
BL53	F	53	4
BL53	F	53	37
BL24	M	63	4
BL24	M	63	37
BL30	M	67	4
BL30	M	67	37
BL32	F	62	4
BL32	F	62	37
BL38	M	61	4
BL38	M	61	37
BL51	F	66	4
BL51	F	66	37
BL36	M	74	4
BL36	M	74	37
BL45	M	76	4
BL45	M	76	37
BL50	F	73	4
BL50	F	73	37
BL56	M	72	4
BL56	M	72	37
BL39	M	79	4
BL39	M	79	37
BL2	F	81	4
BL2	F	81	37
BL54	M	82	4
BL54	M	82	37

Table S1C. Donors' Sex and Age
(CD4+ T cells were incubated in RPMI or AIM V medium for 4h at 37°C and 4°C)

Donor #	Sex	Age	Degree	Medium
ND672	M	30	4	RPMI
ND672	M	30	4	AIM
ND672	M	30	37	RPMI
ND672	M	30	37	AIM
ND561	M	36	4	RPMI
ND561	M	36	4	AIM
ND561	M	36	37	RPMI
ND561	M	36	37	AIM
ND651	M	25	4	RPMI
ND651	M	25	4	AIM
ND651	M	25	37	RPMI
ND651	M	25	37	AIM
ND656	M	32	4	RPMI
ND656	M	32	4	AIM
ND656	M	32	37	RPMI
ND656	M	32	37	AIM
ND229	F	34	4	RPMI
ND229	F	34	4	AIM
ND229	F	34	37	RPMI
ND229	F	34	37	AIM
BL61	M	66	4	RPMI
BL61	M	66	4	AIM
BL61	M	66	37	RPMI
BL61	M	66	37	AIM
BL62	F	64	4	RPMI
BL62	F	64	4	AIM
BL62	F	64	37	RPMI
BL62	F	64	37	AIM
BL63	M	75	4	RPMI
BL63	M	75	4	AIM
BL63	M	75	37	RPMI
BL63	M	75	37	AIM

RPMI 1640 with serum (10% FBS)
AIM V Serum Free Media (no serum)

Table S2. Significant genes in untreated cells (0h).

Gene Symbol	Genbank Accession Number	0O-0Y (Z-ratio)	0O-0Y (p)	0O-0Y (fold change)
CD9	NM_001769.2	6.53	0.0020	1.45
IL1B	NM_000576.2	6.46	0.0391	1.62
C15orf48	NM_032413.2	5.88	0.0196	1.28
LILRA3	NM_006865.2	5.63	0.0270	1.51
OLR1	NM_002543.2	5.51	0.0426	1.17
ENG	NM_000118.1	4.96	0.0233	1.45
CLEC5A	NM_013252.2	4.86	0.0260	1.35
TMEM51	NM_018022.1	4.84	0.0262	1.36
CD14	NM_000591.1	4.47	0.0439	1.31
CST7	NM_003650.2	4.41	0.0497	1.42
PLEK	NM_002664.1	4.39	0.0221	1.31
PTPNS1	NM_080792.1	4.36	0.0219	1.25
CXCL16	NM_022059.1	4.20	0.0198	1.20
FPR1	NM_002029.3	4.12	0.0203	1.29
TNFAIP2	NM_006291.2	4.07	0.0461	1.40
ZNF218	NM_173485.2	4.03	0.0078	1.32
IGSF6	NM_005849.1	4.03	0.0465	1.23
FGR	NM_005248.1	3.93	0.0493	1.34
LYN	NM_002350.1	3.82	0.0279	1.21
SLC16A3	NM_004207.1	3.74	0.0045	1.33
WARS	NM_213646.1	3.73	0.0258	1.32
VASH1	NM_014909.2	3.66	0.0200	1.36
SRXN1	NM_080725.1	3.60	0.0416	1.36
SLC43A2	NM_152346.1	3.52	0.0217	1.22
CTSZ	NM_001336.2	3.48	0.0492	1.31
PLAUR	NM_001005376.1	3.48	0.0094	1.30
MARCKSL1	NM_023009.4	3.44	0.0273	1.21
PEA15	NM_003768.2	3.28	0.0176	1.12
CENTA2	NM_018404.1	3.27	0.0378	1.22
PFC	NM_002621.1	3.23	0.0002	1.29
RAB31	NM_006868.2	3.18	0.0444	1.22
CSTB	NM_000100.2	3.14	0.0253	1.17
HLA-DRB4	NM_021983.4	3.07	0.0329	1.16
C15orf39	NM_015492.3	3.02	0.0313	1.29
CYP27A1	NM_000784.2	2.99	0.0398	1.24
CEBPA	NM_004364.2	2.85	0.0308	1.29
ZDHHC7	NM_017740.1	2.80	0.0422	1.21
UBL5	NM_024292.2	2.78	0.0355	1.18
TAP1	NM_000593.5	2.75	0.0446	1.13
P2RX7	NM_002562.4	2.69	0.0434	1.27
GRINA	NM_000837.1	2.68	0.0371	1.25
CTSB	NM_001908.3	2.64	0.0246	1.20

OR5P3	NM_153445.1	2.61	0.0313	1.28
GSTO1	NM_004832.1	2.60	0.0499	1.15
FGF14	NM_175929.1	2.59	0.0403	1.25
ATP6V1F	NM_004231.2	2.55	0.0195	1.20
LGMN	NM_005606.5	2.51	0.0276	1.21
ZMYND15	NM_032265.1	2.48	0.0283	1.25
STARD8	NM_014725.2	2.42	0.0240	1.28
IFNGR2	NM_005534.2	2.42	0.0489	1.14
ABI3	NM_016428.2	2.32	0.0293	1.28
LITAF	NM_004862.2	2.30	0.0188	1.07
TRIM21	NM_003141.3	2.27	0.0386	1.22
NEUROG3	NM_020999.2	2.20	0.0048	1.23
CMIP	NM_030629.1	2.20	0.0119	1.11
LAIR2	NM_002288.3	2.17	0.0467	1.27
DNCL1	NM_003746.1	2.17	0.0259	1.10
TAPBP	NM_172208.1	2.16	0.0270	1.19
RHOB	NM_004040.2	2.15	0.0463	1.20
PSMA7	NM_152255.1	2.15	0.0424	1.19
SH2D2A	NM_003975.2	2.15	0.0443	1.10
SHKBP1	NM_138392.1	2.13	0.0340	1.23
D15Wsu75e	NM_015704.1	2.11	0.0114	1.20
CENTA1	NM_006869.1	2.10	0.0385	1.25
PAPPA2	NM_020318.1	2.08	0.0323	1.23
TICAM1	NM_014261.1	2.07	0.0281	1.15
CD58	NM_001779.1	2.05	0.0274	1.14
RHOC	NM_175744.3	2.03	0.0135	1.18
C9orf89	NM_032310.2	2.01	0.0455	1.17
PRKCD	NM_212539.1	2.01	0.0143	1.13
C9orf19	NM_022343.2	2.01	0.0264	1.09
LCE1F	NM_178354.1	1.98	0.0024	1.22
MYO1C	NM_033375.3	1.94	0.0445	1.25
TPST2	NM_001008566.1	1.92	0.0014	1.22
MTA2	NM_004739.2	1.91	0.0253	1.15
SAT	NM_002970.1	1.90	0.0468	1.02
CDC42EP2	NM_006779.2	1.89	0.0107	1.22
NT5DC2	NM_022908.1	1.86	0.0040	1.22
MRO	NM_031939.2	1.86	0.0030	1.19
TMEM16G	NM_001001891.1	1.85	0.0175	1.22
LOC94431	NM_145237.1	1.84	0.0067	1.20
CYP4A11	NM_000778.2	1.84	0.0126	1.20
PGD	NM_002631.2	1.84	0.0394	1.14
TNIP3	NM_024873.2	1.83	0.0068	1.23
SPTAN1	NM_003127.1	1.82	0.0398	1.07
SERF1B	NM_022978.1	1.80	0.0426	1.15
ZYX	NM_003461.4	1.80	0.0462	1.09
PSMA5	NM_002790.2	1.79	0.0494	1.08
RNASE1	NM_198232.1	1.77	0.0273	1.21
TA-NFKBH	NM_139239.1	1.77	0.0003	1.20
IGFBP4	NM_001552.2	1.75	0.0481	1.22
C1QA	NM_015991.1	1.70	0.0435	1.22
HMBOX1	NM_024567.2	1.70	0.0414	1.18
RLN2	NM_005059.2	1.69	0.0402	1.22

CLTB	NM_007097.2	1.69	0.0033	1.11
PTPN1	NM_002827.2	1.66	0.0223	1.08
LTBP1	NM_206943.1	1.65	0.0128	1.19
PSMD4	NM_002810.1	1.65	0.0266	1.10
OR2D3	NM_001004684.1	1.61	0.0060	1.18
MTHFD1L	NM_015440.3	1.61	0.0142	1.18
ARPC5L	NM_030978.1	1.61	0.0260	1.07
SP8	NM_198956.1	1.60	0.0231	1.17
SORBS2	NM_003603.4	1.59	0.0338	1.20
LGALS12	NM_033101.2	1.58	0.0278	1.18
DBNL	NM_014063.5	1.58	0.0328	1.06
SIGLEC9	NM_014441.1	1.57	0.0146	1.20
TRPV5	NM_019841.3	1.57	0.0036	1.18
DXS9879E	NM_006014.2	1.56	0.0332	1.12
RPA3	NM_002947.3	1.56	0.0408	1.09
PSORS1C2	NM_014069.1	1.55	0.0149	1.18
DGCR8	NM_022720.5	1.55	0.0070	1.17
GTPBP2	NM_019096.3	1.54	0.0002	1.19
PRY	NM_004676.2	1.54	0.0058	1.18
CD2BP2	NM_006110.1	1.54	0.0219	1.13
BRI3	NM_015379.3	1.54	0.0477	1.09
SR-A1	NM_021228.1	1.53	0.0364	1.20
PVRL2	NM_002856.1	1.53	0.0174	1.18
C19orf30	NM_174947.2	1.51	0.0178	1.16
CBX7	NM_175709.2	1.50	0.0317	1.09
ZNF75A	NM_153028.1	-1.51	0.0446	-1.05
DNAJB14	NM_001031723.1	-1.52	0.0097	-1.07
CHD6	NM_032221.3	-1.53	0.0452	-1.06
B3GALT2	NM_003783.2	-1.54	0.0189	-1.04
JMY	NM_152405.1	-1.54	0.0158	-1.07
PDHX	NM_003477.1	-1.55	0.0356	-1.08
C1orf123	NM_017887.1	-1.55	0.0349	-1.10
NIN	NM_020921.2	-1.56	0.0457	-1.08
GTF2IRD2B	NM_001003795.1	-1.57	0.0382	-1.05
SNTB1	NM_021021.2	-1.57	0.0451	-1.06
C13orf10	NM_022118.3	-1.57	0.0173	-1.11
ZFP1	NM_153688.1	-1.58	0.0195	-1.04
ADK	NM_001123.2	-1.58	0.0220	-1.06
ZNF600	NM_198457.1	-1.58	0.0443	-1.06
ZF	NM_021212.1	-1.58	0.0389	-1.09
PRKRIP1	NM_024653.1	-1.58	0.0161	-1.10
TCEAL8	NM_153333.2	-1.59	0.0078	-1.07
SPHAR	NM_006542.2	-1.60	0.0289	-1.04
MOBK1B	NM_018221.1	-1.60	0.0042	-1.04
BET1L	NM_016526.3	-1.60	0.0234	-1.15
ZNF652	NM_014897.1	-1.61	0.0095	-1.05
C20orf7	NM_199052.1	-1.61	0.0316	-1.09
TUBGCP5	NM_052903.2	-1.62	0.0087	-1.07
USP15	NM_006313.1	-1.62	0.0288	-1.07
AHCTF1	NM_015446.3	-1.63	0.0286	-1.11
C11orf46	NM_152316.1	-1.63	0.0365	-1.11
SMARCA3	NM_003071.2	-1.65	0.0152	-1.07

USP33	NM_201626.1	-1.65	0.0389	-1.12
SETD6	NM_024860.1	-1.66	0.0020	-1.07
NUCKS1	NM_022731.2	-1.66	0.0084	-1.08
FAM3C	NM_014888.1	-1.68	0.0253	-1.06
NIPBL	NM_015384.3	-1.69	0.0374	-1.09
C13orf24	NM_006346.2	-1.71	0.0143	-1.07
CCR9	NM_031200.1	-1.71	0.0010	-1.08
HSU79303	NM_013301.1	-1.72	0.0211	-1.08
POMT1	NM_007171.2	-1.72	0.0137	-1.08
C20orf108	NM_080821.1	-1.72	0.0332	-1.10
ZNF42	NM_198055.1	-1.72	0.0307	-1.11
PPOX	NM_000309.2	-1.75	0.0061	-1.09
PPA2	NM_176869.1	-1.75	0.0332	-1.11
CRSP7	NM_004831.3	-1.75	0.0398	-1.13
SNX25	NM_031953.2	-1.76	0.0335	-1.09
RAB11FIP2	NM_014904.1	-1.76	0.0417	-1.09
KIAA0195	NM_014738.2	-1.76	0.0478	-1.14
ZNF518	NM_014803.2	-1.77	0.0381	-1.10
FLJ30596	NM_153013.2	-1.78	0.0032	-1.06
FLJ22555	NM_024520.1	-1.78	0.0342	-1.13
NR1D2	NM_005126.2	-1.79	0.0398	-1.09
POLR3B	NM_018082.3	-1.80	0.0091	-1.09
KIAA1324L	NM_152748.2	-1.80	0.0323	-1.09
ZNF181	NM_001029997.1	-1.81	0.0426	-1.08
ARG2	NM_001172.3	-1.81	0.0069	-1.08
C10orf35	NM_145306.1	-1.82	0.0053	-1.09
RUNX2	NM_004348.3	-1.82	0.0469	-1.10
TAF1L	NM_153809.1	-1.85	0.0436	-1.07
AMMECR1	NM_001025580.1	-1.85	0.0076	-1.11
ALS2CR13	NM_173511.2	-1.85	0.0476	-1.18
TUBGCP3	NM_006322.3	-1.86	0.0407	-1.08
GOPC	NM_020399.2	-1.86	0.0446	-1.10
INPP5E	NM_019892.3	-1.86	0.0414	-1.14
NT5E	NM_002526.1	-1.87	0.0054	-1.07
C1orf86	NM_182533.1	-1.87	0.0073	-1.09
PIGN	NM_012327.3	-1.87	0.0364	-1.10
RPS6KA5	NM_004755.2	-1.87	0.0163	-1.12
ITM2B	NM_021999.2	-1.87	0.0459	-1.26
NCKAP1	NM_013436.3	-1.88	0.0409	-1.13
GUF1	NM_021927.1	-1.89	0.0178	-1.09
CEP57	NM_014679.3	-1.89	0.0138	-1.10
INPP5A	NM_005539.2	-1.90	0.0219	-1.09
C10orf38	NM_001010924.1	-1.90	0.0118	-1.10
C1QDC1	NM_023925.3	-1.91	0.0211	-1.10
SLC35B3	NM_015948.2	-1.91	0.0382	-1.10
ACAD8	NM_014384.1	-1.92	0.0489	-1.09
KLHL3	NM_017415.1	-1.94	0.0193	-1.12
CARD8	NM_014959.1	-1.95	0.0233	-1.14
MGC3207	NM_032285.1	-1.95	0.0238	-1.15
RNF138	NM_016271.3	-1.97	0.0323	-1.11
ZBTB20	NM_015642.2	-2.00	0.0176	-1.11
ZNF439	NM_152262.1	-2.02	0.0002	-1.09

SLC16A10	NM_018593.3	-2.02	0.0478	-1.11
IDI1	NM_004508.2	-2.03	0.0170	-1.13
SMAP	NM_014267.3	-2.04	0.0313	-1.09
ZNF684	NM_152373.2	-2.05	0.0329	-1.09
HOP	NM_139212.2	-2.05	0.0380	-1.13
C8orf52	NM_017864.2	-2.06	0.0218	-1.14
GCN5L2	NM_021078.1	-2.06	0.0479	-1.16
CAMK2G	NM_172169.1	-2.07	0.0397	-1.11
CARD11	NM_032415.2	-2.07	0.0316	-1.18
ZNF154	NM_003444.1	-2.10	0.0239	-1.11
ZNF644	NM_016620.2	-2.11	0.0144	-1.11
ZNRF2	NM_147128.3	-2.13	0.0346	-1.11
MTX3	NM_001010891.1	-2.13	0.0364	-1.13
FKBP11	NM_016594.1	-2.13	0.0293	-1.20
ST13	NM_003932.3	-2.14	0.0411	-1.16
CNAP1	NM_014865.2	-2.15	0.0025	-1.12
GOLGA8A	NM_181077.2	-2.16	0.0182	-1.11
CROP	NM_006107.2	-2.17	0.0497	-1.14
MFGE8	NM_005928.1	-2.18	0.0303	-1.18
WDR26	NM_025160.4	-2.26	0.0485	-1.19
PLCL1	NM_006226.1	-2.28	0.0036	-1.11
PPIA	NM_203430.1	-2.28	0.0239	-1.14
NARG1L	NM_018527.2	-2.35	0.0244	-1.15
EVA1	NM_144765.1	-2.37	0.0069	-1.13
OFD1	NM_003611.1	-2.38	0.0219	-1.18
PNN	NM_002687.2	-2.41	0.0158	-1.23
SESN1	NM_014454.1	-2.42	0.0225	-1.21
BACH2	NM_021813.1	-2.44	0.0433	-1.17
FLJ34443	NM_175918.2	-2.44	0.0138	-1.19
EIF2AK4	NM_001013703.2	-2.44	0.0409	-1.22
CRSP3	NM_004830.2	-2.46	0.0451	-1.16
IRS2	NM_003749.2	-2.49	0.0103	-1.19
MAP4K5	NM_006575.3	-2.50	0.0284	-1.18
C14orf106	NM_018353.3	-2.51	0.0379	-1.21
TTC14	NM_133462.1	-2.53	0.0144	-1.22
IL11RA	NM_147162.1	-2.54	0.0141	-1.19
LOC159090	NM_145284.3	-2.56	0.0211	-1.15
Ells1	NM_152793.1	-2.57	0.0245	-1.19
ITGA4	NM_000885.4	-2.59	0.0440	-1.21
FAM63A	NM_018379.2	-2.60	0.0324	-1.29
LASS6	NM_203463.1	-2.62	0.0079	-1.20
FBXL3	NM_012158.1	-2.63	0.0319	-1.21
CCNL1	NM_020307.2	-2.64	0.0426	-1.17
HMGB1	NM_002128.3	-2.69	0.0340	-1.13
YPEL2	NM_001005404.3	-2.82	0.0035	-1.21
ABCB1	NM_000927.3	-2.84	0.0246	-1.22
LRMP	NM_006152.2	-2.84	0.0104	-1.23
ADI1	NM_018269.1	-3.02	0.0017	-1.29
LMOD3	NM_198271.2	-3.11	0.0252	-1.35
AKR1C3	NM_003739.4	-3.14	0.0071	-1.28
ITM2A	NM_004867.3	-3.14	0.0138	-1.29
SMAD5	NM_001001419.1	-3.18	0.0178	-1.25

**LYCAT**	**NM_182551.3**	**-3.23**	**0.0182**	**-1.26**
**RPL7**	**NM_000971.3**	**-3.23**	**0.0273**	**-1.32**
**TDRD1**	**NM_198795.1**	**-3.27**	**0.0128**	**-1.28**
**PABPC3**	**NM_030979.2**	**-3.33**	**0.0126**	**-1.22**
**GPR15**	**NM_005290.1**	**-3.35**	**0.0012**	**-1.27**
**RPLP1**	**NM_001003.2**	**-3.37**	**0.0280**	**-1.35**
**ADCY7**	**NM_001114.2**	**-3.48**	**0.0448**	**-1.28**
**BTBD15**	**NM_014155.2**	**-3.50**	**0.0429**	**-1.26**
**ZDHHC11**	**NM_024786.1**	**-3.67**	**0.0423**	**-1.36**
**HBA1**	**NM_000558.3**	**-3.70**	**0.0379**	**-1.45**
**LRAP**	**NM_022350.1**	**-4.35**	**0.0386**	**-1.42**
**HBB**	**NM_000518.4**	**-4.37**	**0.0235**	**-1.60**
**SH3YL1**	**NM_015677.1**	**-4.46**	**0.0281**	**-1.38**
**LRRN3**	**NM_018334.3**	**-8.97**	**0.0000**	**-2.06**

Table S2. Significant genes in untreated cells (0h). Comparison of younger (Y, less than 65 years) and older (O, 65 years or older) cohorts identified with Z-ratios greater than 1.5 and *p*-values less than 0.05. Gene Z-scores within each group were compared between Y and O groups to obtain a Z-ratio indicative of differential expression between the groups. Differentially regulated genes with Z-ratio absolute values greater than 1.5 and *p*-values less than 0.05 are shown in the table arranged by descending Z-ratios. Fold changes, *p*-value and Genebank accession number are as indicated. Positive Z-ratios correspond to genes expressed at higher levels in O compared to Y.

Table S3. Z-ratio of NF-κB-induced pathway genes in first and second group.

Gene Symbol	0O-0Y (group 1) Z-ratio	0O-0Y (group 2) Z-ratio
IL1B	6.464	7.276
CXCL10	5.538	2.826
IL6	4.788	2.209
IL1A	4.293	3.370
TNFAIP2	4.068	3.569
CCL2	4.043	5.635
CXCL2	3.463	2.143
TNF	3.383	1.796
CD83	2.710	2.709
IL8	2.550	4.534
NFKB2	2.388	2.507
MSC	2.308	3.806
CDKN1A	2.202	2.132
SOD2	2.110	2.083
CCL5	1.898	5.136
RELA	1.727	1.738
NFKB1	1.724	2.463
TNFAIP6	1.627	3.855
RELB	1.545	2.735
SPTBN1	-2.001	-3.146

Table S4. Primer sequences used for real-time quantitative PCR

Gene	Forward (F) and Reverse (R) Primers
GAPDH	(F) 5'- TCC ATG ACA ACT TTG GTA TCG TG -3'<br>(R) 5'- ACA GTC TTC TGG GTG GCA GTG -3'
OLR1	(F) 5'- GAA ATC CAA AGA GCA AAT GGA -3'<br>(R) 5'- TTA AAT GAG CCC GAG GAA AA -3'
RELB	(F) 5'- GCC GGC AGC ATC CTT -3'<br>(R) 5'- GAC TCG GTG AGG CCA GTC -3'
CCL20	(F) 5'- GCG CAA ATC CAA AAC AGA CT -3'<br>(R) 5'- ACC TCC AAC CCC AGC AA -3'
IL1 β	(F) 5'- CAA AAT ACC TGT GGC CTT GG -3'<br>(R) 5'- TGA AGA CAA ATC GCT TTT CCA -3'
TNFAIP6	(F) 5'- GGA TTT GGA AAA ACT GGC ATT -3'<br>(R) 5'- TTT GGG AAG CCT GGA GAT TT -3'
